# A Lattice-Based Homomorphic Proxy Re-Encryption Scheme with Strong Anti-Collusion for Cloud Computing

**DOI:** 10.3390/s21010288

**Published:** 2021-01-04

**Authors:** Juyan Li, Zhiqi Qiao, Kejia Zhang, Chen Cui

**Affiliations:** 1 College of Data Science and Technology, Heilongjiang University, Harbin 150080, China; 2018043@hlju.edu.cn (J.L.); qiaozhiqi98@163.com (Z.Q.); zhangkejia@hlju.edu.cn (K.Z.); 2 Guangxi Key Laboratory of Cryptography and Information Security, Guilin 541004, China

**Keywords:** LWE, homomorphic proxy re-encryption, strong anti-collusion, key switching, trapdoor sampling, cloud computing

## Abstract

The homomorphic proxy re-encryption scheme combines the characteristics of a homomorphic encryption scheme and proxy re-encryption scheme. The proxy can not only convert a ciphertext of the delegator into a ciphertext of the delegatee, but also can homomorphically calculate the original ciphertext and re-encryption ciphertext belonging to the same user, so it is especially suitable for cloud computing. Yin et al. put forward the concept of a strong collusion attack on a proxy re-encryption scheme, and carried out a strong collusion attack on the scheme through an example. The existing homomorphic proxy re-encryption schemes use key switching algorithms to generate re-encryption keys, so it can not resist strong collusion attack. In this paper, we construct the first lattice-based homomorphic proxy re-encryption scheme with strong anti-collusion (HPRE-SAC). Firstly, algorithm TrapGen is used to generate an encryption key and trapdoor, then trapdoor sampling is used to generate a decryption key and re-encryption key, respectively. Finally, in order to ensure the homomorphism of ciphertext, a key switching algorithm is only used to generate the evaluation key. Compared with the existing homomorphic proxy re-encryption schemes, our HPRE-SAC scheme not only can resist strong collusion attacks, but also has smaller parameters.

## 1. Introduction

Lattice-based cryptography is a kind of public key cryptosystem, which is widely believed to resist quantum computer attacks. The lattice-based cryptographic systems have attracted the attention of many scholars, on the one hand, because of the simper linear operation than the power operation that is needed in the traditional theory-based cryptosystems (such as RSA); on the other hand, because their security can be based on worst-case hard problems (such as SIVP, GapSVP). There are two basic average-case problems that had been shown to enjoy worst-case hardness guarantee. One is the learning with error (LWE) problem [1,2] the other one is the small integer solution (SIS) problem [3].

Public-key encryption (PKE) is one of the most fundamental primitives in cryptography. In recent years, some lattice-based PKE schemes were constructed based on LWE and SIS [4,5,6]. Fully-homomorphic encryption (FHE) is a kind of PKE, but the FHE scheme allows one to compute arbitrary functions over encrypted data without the decryption key. In an FHE scheme, the data owner can obtain ciphertexts $E\left(m_{1}\right),\cdots ,E\left(m_{n}\right)$ that encrypts data $m_{1},\cdots ,m_{n}$ by encryption key pk (the corresponding decryption key is sk), respectively. Anyone can efficiently compute compact ciphertext that encrypts $f\left(m_{1},\cdots ,m_{n}\right)$ for any efficiently computable function *f*, but only the owner of decryption key sk can get $f\left(m_{1},\cdots ,m_{n}\right)$ by decrypting the compact ciphertext [7,8]. The interesting property makes FHE more applicable in many scenarios, such as cloud computing [9,10].

**Figure 1:**
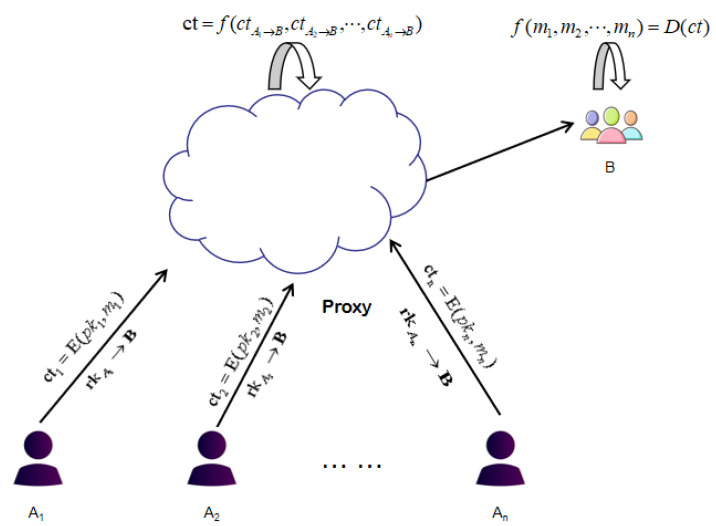
The homomorphic proxy re-encryption scheme.

With the emerging of the cloud computing, the situation has transformed from a single user to multiple users on one of both communication ends. Most of the existing FHE schemes only allow the user to homomorphically compute ciphertexts that are encrypted by himself. Proxy re-encryption (PRE) [11] is an extension of public key encryption. In a PRE scheme, with the help of the re-encryption key, the proxy can convert the ciphertext of a delegator into the ciphertext of a delegatee. In this process, there is no need to decrypt the ciphertext of the delegator, and the proxy can not get the plaintext. It is very interesting to construct a homomorphic proxy re-encryption (HPRE) scheme, which allows users to homomorphically compute ciphertexts of different users. As shown in Figure 1. After getting the ciphertext $ct_{i}=E\left(pk_{i},m_{i}\right)$ and the re-encryption key $rk_{A_{i}\to B}$ of $A_{i},i=1,2,\cdots ,n$, the proxy can convert the ciphertext $ct_{i}$ into the ciphertext $ct_{A_{i}\to B}$ of B, and guarantee the homomorphism of these re-encryption ciphertexts. That is, if $ct=f(ct_{A_{1}\to B},ct_{A_{2}\to B},\cdots ,ct_{A_{n}\to B})$, then $D\left(sk_{B},ct\right)=f\left(m_{1},m_{2},\cdots ,m_{n}\right)$, where $pk_{i}$ is the encryption key of $A_{i}$, $sk_{B}$ is the decryption key of *B*, *f* is an efficiently computable function.

### 1.1. Related Work

Proxy Re-Encryption (PRE) was introduced by Bleumer et al. [11], which can be applied in many scenarios, such as encrypted email forwarding, vehicular ad hoc network, the distributed file system [12], and the cloud sharing [13,14,15,16,17]. Many PRE schemes with special properties have been constructed to meet the increasingly complex cloud sharing environment. For example, conditional proxy re-encryption [18,19], which allows only the ciphertexts satisfying a condition to be converted by the proxy; attribute-based proxy re-encryption [20,21], which transforms a ciphertext under an access policy to a ciphertext under another access policy; broadcast proxy re-encryption [22,23], which converts a ciphertext to a set of ciphertexts under different users at a time; unidirectional proxy re-encryption [24,25], in which the proxy can use the re-encryption key to convert the delegator’s ciphertext to the delegatee’s ciphertext, but cannot reverse the conversion, otherwise it becomes bidirectional; multi-hop proxy re-encryption [26,27], in which the proxy can convert a re-encryption ciphertext into a re-encryption ciphertext of other users, otherwise it becomes single-hop; homomorphic proxy re-encryption (HPRE) scheme [19,28], and so on.

Security is an important index of the practicability of a PRE scheme. At present, the security of a PRE scheme mainly involves post quantum security, semantic security, key privacy, anti-collusion and so on. The construction of PRE can be based on the Diffie–Hellman assumption, but the Diffie-Hellman assumption is not considered post quantum secure. Therefore, it is necessary to construct a PRE based on LWE, because the LWE assumption is generally considered to be able to resist quantum computing attacks. Xagawa [29] constructs the first PRE based on LWE, but the scheme lacks a complete security analysis, and it is bidirectional and can not resist collusion attack. Compared with bidirectional PRE, unidirectional PRE is more in line with the security requirements of cloud sharing. Collusion attack means that the delegatee and the proxy can conspire to compute the decryption key of the delegator.

Aono et al. [30] constructed a unidirectional re-encryption scheme based on LWE and proved that the scheme has key privacy. Key privacy [31] means that even if an active proxy colludes with a set of malicious users in the system, it can not know the identity of the participants involved or the content of their encrypted messages from the re-encryption key. Singh et al. [32] pointed out that the scheme of Aono et al. [30] could not resist collusion attack, and constructed a PRE scheme against collusion attack based on [30]. Kirshanova [33] constructed the first chosen ciphertext attack (CCA) secure lattice-based PRE scheme. Nishimaki et al. [34] constructed two unidirectional single-hop key privacy PRE schemes based on LWE and proved the two schemes are chosen plaintext attack (CPA) secure. Hou et al. [35] constructed an efficient identity-based PRE over lattice and proved that the scheme is CPA secure in the standard model, but the scheme is bidirectional and cannot resist collusion attack. Yin et al. [36] constructed a unidirectional identity based PRE under LWE, and proved that the scheme is CPA secure in the standard model. Yin et al. [37] put forward the concept of a strong collusion attack (the strong collusion attack will be shown in Definition 7) relative to a traditional collusion attack, and called it a traditional collusion attack as weak collusion attack. Yin et al. pointed out through examples that if the adversary can not collude to attack the decryption key of the delegator, but can obtain an approximate value of the decryption key of the delegator, then it can also launch a strong collusion attack on the scheme of Aono et al. [30] and correctly decrypt the ciphertext of the delegator.

Zhong et al. [38] constructed a many-to-one homomorphic encryption scheme based on an approximate GCD problem, which can apply homomorphic addition and homomorphic multiplication to multi-party ciphertexts. However, the scheme is not a lattice-based scheme. Since its introduction, FHE [7,8] has attracted much attention and some FHE schemes have been constructed based on LWE. Since the noise is added at encryption for security, the noise will increase with every homomorphic operation in the FHE scheme based on LWE. For correct decryption, the magnitude of final noise must be less than some bound. How to control noise is an important issue. A number of techniques are proposed and used to control noise growth for building an FHE scheme based on LWE, for example, Brakerski et al. [39] proposed the re-linearization technique and the dimension modulus reduction technique; Brakerski et al. [40] proposed the modulus switching algorithm, Brakerski [41] proposed the scale-invariant technique; Gentry et al. [42] proposed the approximate eigenvector method. In addition, these techniques are also the main techniques for constructing homomorphic proxy re-encryption schemes to control noise growth.

Jiang et al. [26] based on [43] constructed a multi-hop unidirectional lattice-based proxy re-encryption. The scheme can only support one multiplicative homomorphic operation. Ma et al. [19,28] based on [42] constructed a single-hop homomorphic proxy re-encryption from lattices, which allows a user to homomorphically evaluate the original ciphertexts and the re-encrypted ciphertexts, which can come from different users. Li et al. [44,45] constructed a single-hop homomorphic proxy re-encryption via key homomorphic computation and obtained a multi-hop proxy re-encryption using a branching program. Li et al. [46] based on [47] constructed a homomorphic proxy re-encryption from a lattice, which is more flexible than [19,28]. All of these HPR schemes are CPA secure and can not resist strong collusion attack. For the sake of comparison, the comparison results are given in Table 1, which shows the comparison of these PRE schemes in LWE assumption, semantic security, multi-hop, unidirectional-direction (uni-direction), homomorphic encryption (HE) and strong anti-collusion. In this paper, we will construct a lattice-based homomorphic proxy re-encryption scheme with strong anti-collusion. Table 1 shows that our scheme meets all the above performance.

### 1.2. Our Contribution

At present, there are two main methods to construct the re-encryption key in the lattice-based proxy re-encryption scheme. One is to use the key switching algorithm (see Lemma 6) and the other is to use trapdoor sampling technology (see Lemma 3). In fact, the key switching algorithm uses the delegatee’s encryption key to encrypt the delegator’s decryption key and hides the decryption key by noise. Therefore, when the delegatee colludes with the proxy, an approximate value of the delegator’s decryption key can be recovered, that is, the sum of the decryption key and the decryption noise. Thus, the re-encryption key constructed by the key switching algorithm can only resist weak collusion attack, but not strong collusion attack. However, trapdoor sampling technology does not allow inverse operation, that is, we can not get $T_{A}$ or approximate value of $T_{A}$ by $\vec{x},A,\vec{u},\sigma ,\vec{c}$, where $\vec{x}\leftarrow$ SamplePre($A,T_{A},\vec{u},\sigma ,\vec{c}$), so it can resist strong collusion attack.

Because HPRE schemes need to be constructed based on basic homomorphic encryption schemes, and lattice based on homomorphic encryption schemes mostly use a key switching algorithm, modulus switching technique and approximate eigenvector method to control the growth of homomorphic multiplication ciphertext noise, so the current HPRE [28,44,45,46] schemes are constructed based on a key switching algorithm to generate a re-encryption key. The key switching algorithm can not only generate a re-encryption key, but also ensure the homomorphism of ciphertext. However, the re-encryption key generated by key switching algorithm can only resist weak anti-collusion, but not strong anti-collusion. However, the re-encryption key generated by trapdoor sampling technology can resist strong anti-collusion, but it cannot satisfy the homomorphism of ciphertext. This is the difficulty of the HPRE scheme with strong anti-collusion constructed in this paper. Therefore, it is necessary to use trapdoor sampling technology to generate a re-encryption key satisfying the homomorphism of the ciphertext.

In this paper, the ciphertext is divided into two parts, one of which is used to encrypt the plaintext, while ensuring the homomorphism of the ciphertext. In the other part of ciphertext, trapdoor sampling technology can be used to generate the re-encryption key. Therefore, it is necessary to modify the existing homomorphic encryption scheme to make the ciphertext meet the above two requirements.

(1) Firstly, we use the trapdoor technology of [48] to modify the scheme of [1] and construct an L-homomorphic encryption scheme.
(2) Then, based on the L-homomorphic encryption scheme proposed in this paper, we construct an HPRE-SAC scheme by using trapdoor sampling technology and a key switching algorithm.
(3) Finally, a direct application of the HPRE-SAC scheme is given, that is, secure computing of personal health records (PHRs) in the cloud.

Compared with the existing HPRE schemes [28,44,45,46], our HPRE-SAC scheme not only can resist the strong collusion attack, but also has smaller parameters. Therefore, it is more suitable for cloud computing scenarios.

### 1.3. Paper Organization

The rest of this paper is organized as follows. Section 2 is preliminaries. Section 3 describes the building blocks. Section 4 describes a *L*- Homomorphic Encryption Scheme. Section 5 describes the HPRE-SAC Scheme. Lastly, our work is concluded in Section 6.

## 2. Preliminaries

We employ some initial notations listed in Table 2 and let $Z_{q}=\left[-q/2,q/2\right)\cap Z$. When *A* is a matrix, let P2(A) be the matrix formed by applying the operation to each column of *A*.

### 2.1. Lattice and Gaussian Distributions

In this section, we introduce the lattice, Gaussian distribution and some properties needed to construct the scheme.

**Definition** **1.** *Let q be a prime, $A\in Z_{q}^{n\times m}$, $\vec{u}\in Z_{q}^{n}$, define:*

$$\begin{array}{c} \Lambda_{q}^{{}^{⊥}}\left(A\right)=\left\{\vec{e}\in Z^{m},s.t.A\vec{e}=0\;\mathrm{mod}\;q\right\} \end{array}$$

$$\begin{array}{c} \Lambda_{q}^{{}^{\vec{u}}}\left(A\right)=\left\{\vec{e}\in Z^{m},s.t.A\vec{e}=\vec{u}\;\mathrm{mod}\;q\right\} \end{array}$$

**Lemma** **1** ([49]). *Let q≥2 and m≥6nlogq>0. There is a probabilistic polynomial-time (PPT) algorithm TrapGen (q,n,m) that outputs matrixes $A\in Z_{q}^{n\times m}$ which is statistically close to uniform in $Z_{q}^{n\times m}$ and $T\in Z^{m\times m}$ which is a basis for $\Lambda_{q}^{⊥}\left(A\right)$ with $∥T∥\leq O\left(nlogq\right)$ and $∥\tilde{T}∥\leq O\left(\sqrt{n\log q}\right)$ (Alwen et al. asserted that the constant hidden in the first O(·) is no more than 20).*

For any positive parameter σ>0, define the Gaussian function on $R^{m}$, centered at $\vec{c}$: $\forall \vec{x}\in R^{m}$,

ρσ,c→=exp(−πx→−c→2−πx−c2σ2σ2).

Let Λ be a discrete subset of $Z^{m}$. Define the discrete Gaussian distribution over Λ as: $\forall \vec{x}\in R^{m}$,

$$D_{\Lambda ,\sigma ,\vec{c}}\left(\vec{x}\right)=\frac{\rho_{s,\vec{c}}\left(\vec{x}\right)}{\rho_{\sigma ,\vec{c}}\left(\Lambda \right)},$$

where $\rho_{\sigma ,\vec{c}}\left(\Lambda \right)=\sum_{\vec{x}\in \Lambda }\rho_{\sigma ,\vec{c}}\left(\vec{x}\right)$.

**Lemma** **2** ([50]). *Let $\vec{x}\leftarrow D_{Z^{m},\sigma }$, then with overwhelming probability ${∥\vec{x}∥}_{2}<\sigma \sqrt{m}$, where σ>0,.*

**Lemma** **3** ([48]). *Let q≥2, $T_{A}$ be a basis for $\Lambda_{q}^{{}^{⊥}}\left(A\right)$, where $\sigma \geq ∥\tilde{T}∥\omega \left(\sqrt{\log m}\right)$, $A\in Z_{q}^{n\times m}$. Then for any $\vec{c}\in R^{m}$ and $\vec{u}\in Z_{q}^{n}$, there is a PPT algorithm SamplePre($A,T_{A},\vec{u},\sigma ,\vec{c}$) that outputs $\vec{x}\in \Lambda_{q}^{\vec{u}}\left(A\right)$ which is statistically close to $D_{\Lambda_{q}^{\;\vec{u}}\left(A\right),\sigma ,\vec{c}}$.*

**Lemma** **4** ([48]). *The algorithm SamplePre($A,T_{A},\vec{u},\sigma ,\vec{c}$) gives a collection of trapdoor one-way functions with preimage sampling, if $ISIS_{q,m,\sigma \sqrt{m}}$ is hard on average. Furthermore, it gives a collection of trapdoor collision-resistant hash functions with preimage sampling, if $ISIS_{q,m,\sigma \sqrt{m}}$ is hard on the average.*

**Definition** **2** ([1]). *Let k be the security parameter, and χ=χ(k) be a distribution over $Z_{q}$. The $\mathrm{LW}E_{n,m,q,\chi }$ assumption shows that, if $A\leftarrow Z_{q}^{m\times n},\vec{s}\leftarrow Z_{q}^{n},\vec{e}\leftarrow \chi^{m},\;\vec{u}\leftarrow Z_{q}^{m}$, then*

$$\left(A,A\vec{s}+\vec{e}\right)\approx_{c}\left(A,\vec{u}\right).$$

It is well known that if $\chi^{m}=D_{Z^{m},\alpha q}$, then when $\alpha q\geq 2\sqrt{n}$, this decision LWE problem is at least as hard as approximating several problems on *n*-dimensional lattices Λ in the worst-case to within O˜(nα) factors with a quantum computer.

### 2.2. HE: Definition and Security

In this section, we show the definition and security model of the homomorphic encryption (HE) scheme based on [41].

**Definition** **3.** *(Homomorphic encryption scheme)*

*A homomorphic encryption scheme consists of the following five algorithms:*

*1.*  *$HE.Setup(1^{k})\to pp:$ Input the security parameter k. Output the public parameters pp.*
*2.*  *HE.KeyGen(pp)→(pk,sk,evk): Input the public parameters pp. Output the encryption key pk, the public evaluation key evk and the decryption key sk.*
*3.*  *HE.Enc(pp,pk,μ)→ct: Input pp,pk, and a message μ∈{0,1}. Output a ciphertext ct.*
*4.*  *$HE.Eval\left(pp,f,ct_{1},\cdots ,ct_{l},evk\right)\to ct_{f}$: Input $pp,ct_{1},\cdots ,ct_{l},evk$ and a function f: ${\left\{0,1\right\}}^{l}\to \left\{0,1\right\}$. Output a ciphertext $ct_{f}$. (We consider homomorphic addition $-Add\left(ct_{1},ct_{2},evk\right)\to {ct}_{add}$ and multiplication $-Mult\left(ct_{1},ct_{2},evk\right)\to {ct}_{mult}$ of depth L arithmetic circuits f over GF(2) in a gate-to-gate manner.)*
*5.*  *HE.Dec(pp,sk,ct)→μ: Input pp,sk and ciphertext ct under secret key sk. Output the message μ.*

Compared with the public key encryption scheme, the adversary obtains not only pk but also evk in the HE scheme. If the homomorphic encryption scheme is still semantically secure when the adversary obtains pk and evk, it is said that the HE scheme is secure. The security model of HE scheme is omitted here.

**Definition** **4.** *(L-homomorphism) If for any depth L=L(k) arithmetic circuit f over GF(2) and any set of inputs $\mu_{1},\cdots ,\mu_{l}\in \left\{0,1\right\}$, it holds that*

$$HE.Dec\left(HE.Eval(pp,f,ct_{1},\cdots ,ct_{l})\right)=f\left(\mu_{1},\cdots ,\mu_{l}\right)$$

*with overwhelming probability of k, where (pk,sk,evk)←HE.KeyGen(pp), $ct_{i}\leftarrow HE.Enc\left(pp,pk,\mu_{i}\right)$. Then the HE scheme is L-homomorphic.*

### 2.3. HPRE: Definition and Security Model

**Figure 2:**
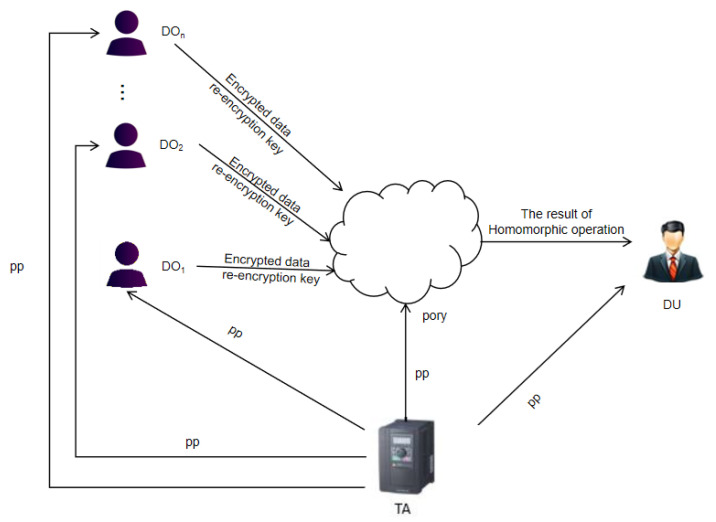
System model of the homomorphic proxy re-encryption (HPRE) scheme.

In this subsection, we recall the definition and the security model of the homomorphic proxy re-encryption (HPRE) scheme. There are four participants in the unidirectional HPRE scheme for cloud sharing, as shown in Figure 2.

(1) Trusted authority (TA). The TA is trusted by all participants. TA generates the public parameters pp.
(2) Proxy. The proxy is semi-trusted by all participants. Proxy is generally a cloud service provider. Users use the cloud service provider to store and calculate data.
(3) Data owner (DO). The DO encrypts the data and stores the encrypted data in the cloud, and generates a proxy re-encryption key for data users.
(4) Data user (DU). The DU downloads the result of the homomorphic operation from the cloud service provider.

**Definition** **5.** *(Unidirectional homomorphic proxy re-encryption scheme )*

*A unidirectional HPRE scheme consists of the following seven algorithms:*

*1.*  *HPRE.Setup$(1^{k},1^{L})\to pp$: For the security parameter k, the upper bound on the maximal multiplicative depth L=L(k) that the scheme can homomorphically evaluate, the TA outputs the public parameters pp.*
*2.*  *HPRE.KeyGen$\left(pp,L\right)\to \left(pk^{i},sk^{i},evk^{i}\right)$: For pp,L, user i (DO or DU) outputs an encryption/decryption key pair $\left(pk^{i},sk^{i}\right)$, and public evaluation key $evk^{i}$.*
*3.*  *HPRE.Enc(pp,pk,μ)→ct: For pp, pk and a message μ, user (DO or DU) outputs an original ciphertext ct.*
*4.*  *HPRE.Rekey$\left(pp,sk^{i},pk^{i},pk^{j}\right)\to rk^{i\to j}$: For pp, an encryption/decryption key pair $\left(pk^{i},sk^{i}\right)$ of user i, and an encryption key $pk^{j}$ of user j, user i outputs a re-encryption key $rk^{i\to j}$.*
*5.*  *HPRE.ReEnc$\left(pp,rk^{i\to j},ct^{i}\right)\to ct^{j}:$ For pp, a re-encryption key $rk^{i\to j}$, and an original ciphertext $ct^{i}$ of user i, the proxy outputs a re-encryption ciphertext $ct^{j}$ for the user j.*
*6.*  *HPRE.Eval$\left(pp,f,ct_{1},\cdots ,ct_{l},evk\right)\to ct_{f}$: For $pp,ct_{1},\cdots ,ct_{l},evk$ and a function f: ${\left\{0,1\right\}}^{l}\to \left\{0,1\right\}$, the proxy outputs a ciphertext $ct_{f}$. (We consider homomorphic addition $-Add\left(ct_{1},ct_{2},evk\right)\to {ct}_{add}$ and multiplication $-Mult\left(ct_{1},ct_{2},evk\right)\to {ct}_{mult}$ of depth L arithmetic circuits f over GF(2) in a gate-to-gate manner. In addition, it should be noted that the ciphertexts $ct_{1},\cdots ,ct_{l}$ belonging to a user can be original ciphertext or re-encryption ciphertext.)*
*7.*  *HPRE.Dec$\left(pp,sk,ct\right)\to \mu$: For pp,sk and a ciphertext ct under sk, user outputs the message μ.*

Now we define the security model of the HPRE scheme.

**Definition** **6.** *Let HPRE=(HPRE.Setup, HPRE.KeyGen, HPRE.Enc, HPRE.Rekey, HPRE.ReEn, HPRE.Eval, HPRE.Dec) be a unidirectional HPRE scheme, k be a security parameter. Consider the following games $Expt_{HPRE,A}^{IND-CPA}\left(k\right)$ between challenger and adversary.*

*Setup Phase 1: Given a security parameter k, the challenger obtains public parameters pp by running HPRE.Setup $\left(1^{k},1^{L}\right)$ and sends pp to adversary.*

*Learning Phase: In this phase, the adversary can issue the queries to the following oracles polynomially many times, and the challenger needs to answer these oracles.*

*Encryption key generation oracle $O_{pk}$: Given a user index i, the challenger obtains $\left(pk^{i},sk^{i},evk^{i}\right)$ of user i by running HPRE.KeyGen(pp,L) which are recorded in a table, and returns $pk^{i}$ to the adversary.*

*Evaluation key generation oracle $O_{evk}$: Given a user index i, the challenger first looks for the table and returns $evk^{i}$ if there is an $evk^{i}$ in the table. Otherwise, the challenger obtains $\left(pk^{i},sk^{i},evk^{i}\right)$ of user i by running HPRE.KeyGen(pp,L), returns $evk^{i}$ to the adversary, and records $\left(pk^{i},sk^{i},evk^{i}\right)$ in the table.*

*Decryption key generation oracle $O_{sk}$: Given a user index i, if user i is an honest user, the challenger returns ⊥. If user i is a corrupted user, the challenger first looks for the table and returns $sk^{i}$ if there is a $sk^{i}$ in the table. Otherwise, the challenger obtains $\left(pk^{i},sk^{i},evk^{i}\right)$ of user i by running HPRE.KeyGen(pp,L), returns $sk^{i}$ to the adversary, and records $\left(pk^{i},sk^{i},evk^{i}\right)$ in the table.*

*Re-encryption key generation oracle $O_{rk}$: Given two user indices (i,j), if user i and user j are honest or corrupted, the challenger obtains $rk^{i\to j}$ by running HPRE.Rekey $\left(pp,sk^{i},pk^{i},pk^{j}\right)$, and returns the $rk^{i\to j}$ to the adversary, where i≠j. Otherwise, the challenger returns* ⊥.

*Re-encryption ciphertext generation oracle $O_{re}$: Given two user indices (i,j) and a ciphertext $ct^{i}$ of user i, if user i and user j are honest or corrupted, the challenger obtains a ciphertext $ct^{j}$ of user j by running HPRE.ReEnc $\left(pp,rk^{i\to j},ct^{i}\right)$ and returns $ct^{j}$ to the adversary, where i≠j, $rk^{i\to j}\leftarrow HPRE.Rekey\left(pp,sk^{i},pk^{i},pk^{j}\right)$. Otherwise, the challenger returns* ⊥.

Challenge: The adversary gives a target honest user $i^{*}$ and a message μ after finishing all queries. The challenger chooses b←{0,1}, computes $ct_{0}^{i*}\leftarrow HPRE.Enc\left(pp,pk,\mu \right)$, lets $ct_{1}^{i*}$ be a random ciphertext, and sends the challenge ciphertext $ct_{b}^{i^{*}}$ to the adversary.

Learning Phase 2: The adversary could ask extra queries that for decryption key query, re-encryption key query and re-encryption query on the $i\neq i^{*}$, the challenger responses are the same as in Learning Phase 1.

*Finalization: Output 1 if $b^{\prime }=b$. Otherwise, output 0.*

*We say a unidirectional HPRE scheme is IND-CPA secure if for any PPT adversary, the advantage*

$$\begin{array}{c} Adv{}_{FHPRE,A}^{IND-CPA}\left(k\right)=\left|\begin{array}{c} \mathrm{Pr}\left[Expt{}_{FHPRE,A}^{IND-CPA}\left(k\right)\to 1\left|b=1\right.\right]\\ \;\;-\mathrm{Pr}\left[Expt{}_{FHPRE,A}^{IND-CPA}\left(k\right)\to 1\left|b=0\right.\right] \end{array}\right| \end{array}$$

*of adversary is negligible in k.*

Yin et al. [37] put forward the concept of strong collusion attack relative to traditional collusion attack, and called traditional collusion attack as weak collusion attack. Yin et al. pointed out through examples that if the adversary can not collude to attack the decryption key of the delegator, but can obtain an approximate value of the decryption key of the delegator, then it can also launch a strong collusion attack on the scheme of Aono et al. [30] and correctly decrypt the ciphertext of the delegator. In fact, the approximate value of the decryption key obtained by the strong collusion attack is P2(S)+X, where *S* is the decryption key of the delegator, and *X* is an error distribution (generally Gaussian distribution). Therefore, an approximate value of *S* can be obtained. Combined with the definition of a strong collusion attack of Yin et al. [37], we give a new definition of strong collusion attack.

**Definition** **7.** *In a unidirectional proxy re-encryption scheme, if the proxy (cloud service provider) and the delegatee (data user) can not collude to obtain the decryption key S or an approximate value P2(S)+X of the decryption key of the delegator (data owner), the scheme is called strong anti-collusion, where X is an error distribution. If the decryption key S can not be calculated by collusion, but the approximate value P2(S)+X of the decryption key can be obtained, it is called weak anti-collusion, where X is an error distribution.*

## 3. Building Blocks

In this section, we construct a new encryption scheme based on [1,48]. Based on this new basic encryption scheme, we can construct a homomorphic proxy re-encryption (HPRE) scheme against strong collusion attack, which is named HPRE-SAC.

### 3.1. The Basic Encryption Scheme

The basic encryption scheme consists of the following four algorithms.

- $E.Setup(1^{k}):$ Input the security parameter *k*, sample $\vec{u}\leftarrow Z_{q}^{n}$. Output the public parameters $pp=(1^{k},1^{n},q,\chi ,\vec{u})$.
- E.KeyGen(pp): Input the public parameters pp, use algorithm TrapGen(q,n,m) to generate matrices $A\in Z_{q}^{n\times m}$ with trapdoor basis *T*, where m≥6nlogq. Then use algorithm SamplePre$\left(A,T,\vec{u}\right)$ to sample a vector $\vec{s}\in Z_{q}^{m}$, where $A\vec{s}=\vec{u}$. Output the encryption key $pk=(\vec{u}|-A)$ and the decryption key $sk=(\vec{s},T)$. (Note that the decryption key *T* is redundant here, we can instead just let $sk=\vec{s}$. The decryption key *T* is needed to construct the PRE scheme, as described below.)
- E.Enc(pp,pk,μ): Input the public parameters pp, the encryption key $pk=(\vec{u}|-A)$ and a message μ∈{0,1}. Output a ciphertext $ct\in Z_{q}^{1\times m+1}$,
  $$ct={\vec{e}}^{t}\left(\vec{u}|-A\right)+{\vec{y}}^{t}+\left\lfloor \frac{q}{2}\right\rfloor {\vec{\mu }}^{t},$$
  where ${\vec{\mu }}^{t}=\left(\mu ,0,\cdots ,0\right)$, $\vec{e}\leftarrow \chi^{1\times n}$, $\vec{y}\leftarrow \chi^{1\times m+1}$.
- E.Dec(pp,sk,ct): Input the public parameters pp, the decryption key $sk=(\vec{s},T)$ and a ciphertext ct. Compute and output
  $$\mu ={\left[\left\lfloor \frac{2}{q}{\left[ct\left(1;\vec{s}\right)\right]}_{q}\right\rceil \right]}_{2}.$$

### 3.2. Correctness Analysis

We show the correctness in this subsection.

For a ciphertext a ciphertext $ct={\vec{e}}^{t}\left(\vec{u}|A\right)+{\vec{y}}^{t}+\left\lfloor \frac{q}{2}\right\rfloor {\vec{\mu }}^{t}$, where $\vec{\mu }=\left(\mu ,0,\cdots ,0\right)$, $\vec{e}\leftarrow \chi^{1\times n}$, $\vec{y}\leftarrow \chi^{1\times m+1}$. We have

$$\begin{array}{cc} ct\left(1;\vec{s}\right) & =\left({\vec{e}}^{t}\left(\vec{u}|-A\right)+{\vec{y}}^{t}+\left\lfloor \frac{q}{2}\right\rfloor {\vec{\mu }}^{t}\right)\left(1;\vec{s}\right)\\ & ={\vec{e}}^{t}\left(\vec{u}|-A\right)\left(1;\vec{s}\right)+{\vec{y}}^{t}\left(1;\vec{s}\right)+\left\lfloor \frac{q}{2}\right\rfloor {\vec{\mu }}^{t}\left(1;\vec{s}\right)\\ & =\underset{x}{\underset{︸}{{\vec{y}}^{t}\left(1;\vec{s}\right)}}+\left\lfloor \frac{q}{2}\right\rfloor \mu . \end{array}$$

If ${∥x∥}_{\infty }<\left\lfloor \frac{q}{2}\right\rfloor /2$, then the decryption is correct.

For the correctness of this scheme, it needs to satisfy the following conditions:

(1) ${∥x∥}_{\infty }={∥{\vec{y}}^{t}\left(1;\vec{s}\right)∥}_{\infty }<\left\lfloor \frac{q}{2}\right\rfloor /2$
(2) Algorithm TrapGen requires m≥6nlogq.
(3) Algorithm SamplePre requires $\sigma \geq ∥\tilde{T}∥\omega \left(\sqrt{\log m}\right)$.

Because $∥T∥<O\left(n\log q\right)$, $∥\vec{y}∥,∥\vec{s}∥\leq \sigma \sqrt{m}$, we set the parameters as follows: n=k, *q*=the prime nearest to $2^{n^{\delta }}$, $m=6n\left\lceil \log q\right\rceil$, $\sigma =m\omega \left(\sqrt{\log m}\right)$, where δ is constant between 0 and 1. So we have the following Lemma 5.

**Lemma** **5.** *Let q,k,m,n be parameters for the above basic encryption scheme, χ be B-bounded. Set $\left(\vec{s},A\right)\leftarrow E.KeyGen\left(pp\right)$, and ct←E.Enc(pp,pk,μ). Then*

$$ct\left(1;\vec{s}\right)=x+\left\lfloor \frac{q}{2}\right\rfloor \mu ,$$

*where ${∥x∥}_{\infty }={∥{\vec{y}}^{t}\left(1;\vec{s}\right)∥}_{\infty }\leq \left(m+1\right)B^{2}$. If $\left(m+1\right)B^{2}<\left\lfloor \frac{q}{2}\right\rfloor /2$, then μ←E.Dec(pp,sk,ct).*

### 3.3. Security Analysis

We now outline the proof of security to show that the scheme is CPA secure based on LWE assumption. Since $\vec{u}\leftarrow Z_{q}^{n}$, and A← TrapGen (q,n,m), we have $\left(\vec{u}|A\right)$ uniformly distributed by Lemma 1. From LWE, we know that ${\vec{e}}^{t}\left(\vec{u}|-A\right)+{\vec{y}}^{t}$ is uniformly distributed and ct hides $\left\lfloor \frac{q}{2}\right\rfloor {\vec{\mu }}^{t}$. Therefore, the basic encryption scheme is IND-CPA secure.

### 3.4. Key Switching

Based on the technology of [41], and the basic encryption scheme, we construct a key switching algorithm, which can switch the ciphertext under the decryption key ${\vec{s}}_{1}\in Z_{q}^{n_{1}}$ into the ciphertext under the decryption key $\left(1;{\vec{s}}_{2}\right)\in Z_{q}^{\left(n_{2}+1\right)}$.

- $SwitchKeyGen({\vec{s}}_{1},{\vec{s}}_{2}):$ Input decryption keys ${\vec{s}}_{1}\in Z_{q}^{n_{1}}$, ${\vec{s}}_{2}\in Z_{q}^{n_{2}}$. Sample $A_{{\vec{s}}_{1}:{\vec{s}}_{2}}\leftarrow Z_{q}^{n_{1}\left\lceil logq\right\rceil \times n_{2}\;}$, ${\vec{x}}_{{\vec{s}}_{1}:{\vec{s}}_{2}}\leftarrow \chi^{n_{1}\left\lceil logq\right\rceil }$, compute
  $${\vec{b}}_{{\vec{s}}_{1}:{\vec{s}}_{2}}=A_{{\vec{s}}_{1}:{\vec{s}}_{2}}{\vec{s}}_{2}+{\vec{x}}_{{\vec{s}}_{1}:{\vec{s}}_{2}}+P2\left({\vec{s}}_{1}\right).$$
  Output a matrix
  $$P_{{\vec{s}}_{1}:{\vec{s}}_{2}}=\left({\vec{b}}_{{\vec{s}}_{1}:{\vec{s}}_{2}}|-A_{{\vec{s}}_{1}:{\vec{s}}_{2}}\right)\in Z_{q}^{n_{1}\left\lceil logq\right\rceil \times \left(1+n_{2}\right)}.$$
- $SwitchKey(P_{{\vec{s}}_{1}:{\vec{s}}_{2}},ct_{S_{1}}):$ Input a ciphertext $ct_{{\vec{s}}_{1}}$ under the decryption key ${\vec{s}}_{1}$, and $P_{{\vec{s}}_{1}:{\vec{s}}_{2}}$. Output a ciphertext
  $$ct_{{\vec{s}}_{2}}=P_{{\vec{s}}_{1}:{\vec{s}}_{2}}^{t}BD\left(ct_{{\vec{s}}_{1}}\right).$$

**Lemma** **6.** *(correctness) Let ${\vec{s}}_{1}\in Z_{q}^{n_{1}},{\vec{s}}_{2}\in Z_{q}^{n_{2}}$. Let $P_{{\vec{s}}_{1}:{\vec{s}}_{2}}\leftarrow SwitchKeyGen\left({\vec{s}}_{1},{\vec{s}}_{2}\right)$ and $ct_{{\vec{s}}_{2}}\leftarrow SwitchKey\left(P_{{\vec{s}}_{1}:{\vec{s}}_{2}},ct_{{\vec{s}}_{1}}\right)$. Then*

$$ct_{{\vec{s}}_{1}}^{t}{\vec{s}}_{1}=ct_{{\vec{s}}_{2}}^{t}\left(1;{\vec{s}}_{2}\right)-BD\left(ct_{{\vec{s}}_{1}}^{t}\right){\vec{x}}_{{\vec{s}}_{1}:{\vec{s}}_{2}}.$$

**Lemma** **7.** *(security) Let ${\vec{s}}_{1}\in Z_{q}^{n_{1}}$ be any vector, if ${\vec{s}}_{2}\in Z_{q}^{n_{2}}\leftarrow E.KeyGen\left(pp\right)$, $P_{{\vec{s}}_{1}:{\vec{s}}_{2}}\leftarrow SwitchKeyGen\left({\vec{s}}_{1},{\vec{s}}_{2}\right)$. Then $P_{{\vec{s}}_{1}:{\vec{s}}_{2}}$ is computationally indistinguishable from uniform over $Z_{q}^{n_{1}\left\lceil logq\right\rceil \times \left(1+n_{2}\right)}$ based on LWE.*

## 4. An L- Homomorphic Encryption Scheme

In this section, we construct an L-homomorphic encryption scheme based on the basic encryption scheme with the help of the technology of [41,47].

### 4.1. Construction

An L- homomorphic encryption scheme consists of the following five algorithms.

- $HE.Setup(1^{L},1^{k})$: Input the security parameter *k*, sample $\vec{u}\leftarrow Z_{q}^{n}$, and let *L* be the maximum depth of arithmetic circuit supporting homomorphic evaluation. Output the public parameters $pp=(1^{k},1^{n},1^{m},q,\chi ,\vec{u},L)$.
- HE.KeyGen(pp): Input the public parameters pp, use algorithm TrapGen(q,n,m) to generate matrices $A\in Z_{q}^{n\times m}$ with trapdoor basis *T*, where m≥6nlogq, use algorithm SamplePre$\left(A,T,\vec{u}\right)$ to sample a vector ${\vec{s}}_{0}\in Z_{q}^{m}$, where $A{\vec{s}}_{0}=\vec{u}$, sample ${\vec{s}}_{l}\leftarrow \chi^{m}$ and compute
  (1) $${\vec{s}}_{l}^{*}=BD\left(1;\vec{s_{l}}\right)⊗BD\left(1;\vec{s_{l}}\right)\in {\left\{0,1\right\}}^{{\left(\left(m+1\right)\left\lceil logq\right\rceil \right)}^{2}},$$
  $$P_{(l-1):l}\leftarrow SwitchKeyGen\left({\vec{s}}_{l-1}^{*},{\vec{s}}_{l}\right),$$
  where l=1,2,⋯,L. Output the encryption key $pk=(\vec{u}|-A)$, the decryption key $sk=({\vec{s}}_{L},T)$, $evk={\left\{P_{(l-1):l}\right\}}_{l=1,2,\cdots ,L}$. (Note that the decryption key *T* is redundant here, we can instead just let $sk={\vec{s}}_{L}$. The decryption key *T* is needed to construct the PRE scheme, as described below.)
- HE.Enc(pp,pk,μ): Identical to the basic encryption scheme, output ct←E.Enc(pp,pk,μ).
- HE.Eval(.): As [41,47], We consider homomorphic addition and multiplication of depth *L* arithmetic circuits over GF(2) in a gate-to-gate manner. That is, the decryption key of the ciphertexts operated by the gate at level i of the circuit is ${\vec{s}}_{i-1}$, and the decryption key of the ciphertexts output by the homomorphic operation is ${\vec{s}}_{i}$.
  $-Add(ct_{1},ct_{2})$: Input ciphertexts $ct_{1},ct_{2}$ under secret key $S_{i-1}$, compute
  (2) $${\tilde{ct}}_{add}=P2\left(ct_{1}+ct_{2}\right)⊗P2\left(1,,0,\cdots ,0\right),$$
  and output
  $${ct}_{add}\leftarrow SwitchKey\left(P_{(l-1):l},{\tilde{ct}}_{add}\right).$$
  $-Mult(ct_{1},ct_{2})$: Input ciphertexts $ct_{1},ct_{2}$ under secret key $S_{i-1}$, compute
  (3) $${\tilde{ct}}_{mult}=\left\lfloor \frac{2}{q}\left(P2\left(ct_{1}\right)⊗P2\left(ct_{2}\right)\right)\right\rceil ,$$
  and output
  $${ct}_{mult}\leftarrow SwitchKey\left(P_{(l-1):l},{\tilde{ct}}_{mult}\right).$$
- HE.Dec(pp,sk,ct): Input ciphertexts ct under secret key ${\vec{s}}_{L}$. Output μ←E.Dec(pp,sk,ct).

### 4.2. Analysis for Homomorphism

We next show the homomorphism of the above L- Homomorphic Encryption scheme.

**Lemma** **8.** *Let q,k,m,n,s,L,χ be parameters for the above homomorphic encryption scheme, χ be B-bounded, and (pk,sk,evk)←HE.KeyGen(pp). Let $ct_{1},ct_{2}$ be such that*

(4) $$\begin{array}{c} ct_{1}\left(1;{\vec{s}}_{l}\right)=x_{1}+\left\lfloor \frac{q}{2}\right\rfloor \mu_{1},\\ ct_{2}\left(1;{\vec{s}}_{l}\right)=x_{2}+\left\lfloor \frac{q}{2}\right\rfloor \mu_{2}, \end{array}$$

*$∥x_{1}{∥}_{\infty },{∥x_{2}∥}_{\infty }\leq E<\left\lfloor \frac{q}{2}\right\rfloor /2$. Set $ct_{add}\leftarrow Add\left(ct_{1},ct_{2}\right)$, $ct_{mult}\leftarrow Mult\left(ct_{1},ct_{2}\right)$, then*

$$ct_{add}\left(1;{\vec{s}}_{l+1}\right)=x_{add}+\left\lfloor \frac{q}{2}\right\rfloor {\left[\mu_{1}+\mu_{2}\right]}_{2},$$

$$ct_{mult}\left(1;{\vec{s}}_{l+1}\right)=x_{mult}+\left\lfloor \frac{q}{2}\right\rfloor \mu_{1}\mu_{2},$$

*where $∥x_{add}{∥}_{\infty }$, $∥x_{mult}{∥}_{\infty }\leq O\left(mlogq\right)\cdot max\left\{\left(mlog^{2}q\right)B,E\right\}$.*

**Theorem** **1.** *Let q,k,m,n,L be parameters for the above HE scheme, χ be B-bounded. If ${\left(O(mlogq)\right)}^{L+O(1)}\leq q/B^{2}$, then the HE scheme is L homomorphic.*

**Proof.**  Let $E_{i}$ be the bound of noise after evaluation on the i−th level of gates in ciphertext. By Lemma 5, we have $E_{0}\leq \left(m+1\right)B^{2}=O\left(m\right)B^{2}$. According to Lemma 8, when $mlog^{2}qB\leq E$ holds at a certain point, then $E_{i+1}=O\left(mlogq\right)\cdot E_{i}$ and $E_{L}={\left(O(mlogq)\right)}^{L+O(1)}\cdot B^{2}$. Therefore, the decryption is correct if $E_{L}<\left\lfloor \frac{q}{2}\right\rfloor /2$, that is ${\left(O(mlogq)\right)}^{L+O(1)}<q/B^{2}$. □

### 4.3. Security Analysis

We now outline the proof of security to show that the HE scheme is CPA secure based on LWE assumption. We show $\left(pk,evk,ct\right)=(\left(\vec{u}|A\right),{\left\{P_{(l-1):l}\right\}}_{l=1,2,\cdots ,L},ct)$ is indistinguishable from uniform by applying a hybrid argument. Since ${\vec{s}}_{L}$ is only used to generate $P_{(L-1):L}$, we can get $P_{(L-1):L}$ is indistinguishable from uniform by Lemma 7. Then we can proceed to replace all $P_{(l-1):l}$ with uniform in descending order. Finally, there is only $\left(\left(\vec{u}|A\right),ct\right)$ left, which is indistinguishable from uniform by the security analysis of the basic encryption scheme.

## 5. The HPRE-SAC Scheme

In this section, we will use the above homomorphic encryption (HE) scheme to construct the HPRE-SAC scheme by using Trapdoor Sampling [27,48].

### 5.1. Construction

The HPRE-SAC scheme consists of the following seven algorithms.

- $HPRE.Setup(1^{k},1^{L}):$ Identical to the HE scheme, output $pp\leftarrow HE.Setup(1^{k},1^{L})$.
- HPRE.KeyGen(pp): Identical to the HE scheme, output (sk,pk,evk)←HE.KeyGen(pp).
- HPRE.Enc(pp,pk,μ): Identical to the HE scheme, output ct←HE.Enc(pp,pk,μ)
- $HPRE.ReKey(pp,sk^{i},pk^{i},pk^{j})$: Input pp, the encryption key $pk^{i}=\left(\vec{u}|-A^{i}\right)$ and the decryption key $sk^{i}=\left({\vec{s}}_{L}^{i},T^{i}\right)$ of user *i*, the encryption key $pk^{j}=\left(\vec{u}|-A^{j}\right)$ of user *j*, sample $X^{i\to j}\leftarrow \chi^{n\times m}$, use algorithm SamplePre($A^{i},T^{i},A^{j}+X^{i\to j}$) to sample a matrix $R^{i\to j}$, where
  (5) $$A^{i}R^{i\to j}=A^{j}+X^{i\to j},$$
  output the re-encryption key $rk^{i\to j}=R^{i\to j}$.
- $HPRE.ReEnc(pp,rk^{i\to j},ct^{i}):$ Input pp, a original ciphertext $ct^{i}$ of user *i*, and a re-encryption key $rk^{i\to j}=R^{i\to j}$. Output a re-encryption ciphertext
  (6) $$ct^{j}=ct^{i}\left[\begin{array}{cc} 1 & 0_{1\times m}\\ 0 & R^{i\to j} \end{array}\right]+{\left({\vec{z}}^{i\to j}\right)}^{t}$$
  for user *j*, where ${\vec{z}}^{i\to j}\leftarrow \chi^{1\times (m+1)}$.
- HPRE.Eval$\left(pp,f,ct_{1},\cdots ,ct_{l},evk\right)\to ct_{f}$: Except for the ciphertexts $ct_{1},\cdots ,ct_{l}$ that belongs to a user can be the original ciphertext or re-encryption ciphertext, the rest are the same as HE scheme, $ct_{f}\leftarrow HE.Eval\left(pp,f,ct_{1},\cdots ,ct_{l},evk\right)$.
- HPRE.Dec$\left(pp,sk,ct\right)\to \mu$: Identical to the HE scheme, output $\mu \leftarrow HE.Dec\left(pp,sk,ct\right)$.

### 5.2. Correctness Analysis

We show the correctness in this subsection.

For a original ciphertext, we know the decryption is correct by Lemma 5. For a re-encryption ciphertext $ct^{j}=ct^{i}\left[\begin{array}{cc} 1 & 0_{1\times m}\\ 0 & R^{i\to j} \end{array}\right]+{\left({\vec{z}}^{i\to j}\right)}^{t}$, where $ct^{i}={\vec{e}}^{it}\left(\vec{u}|-A^{i}\right)+{\vec{y}}^{it}+\left\lfloor \frac{q}{2}\right\rfloor {\vec{\mu }}^{it}$, ${\vec{\mu }}^{it}=\left(\mu ,0,\cdots ,0\right)$, ${\vec{e}}^{i}\leftarrow \chi^{1\times n}$, ${\vec{y}}^{i}\leftarrow \chi^{1\times m+1}$, ${\vec{z}}^{i\to j}\leftarrow \chi^{1\times (m+1)}$, we have

$$\begin{array}{cc} ct^{j} & =ct^{i}\left[\begin{array}{cc} 1 & 0_{1\times m}\\ 0 & R^{i\to j} \end{array}\right]+{\left({\vec{z}}^{i\to j}\right)}^{t}\\ & =\left({\vec{e}}^{it}\left(\vec{u}|-A^{i}\right)+{\vec{y}}^{it}+\left\lfloor \frac{q}{2}\right\rfloor {\vec{\mu }}^{it}\right)\left[\begin{array}{cc} 1 & 0_{1\times m}\\ 0 & R^{i\to j} \end{array}\right]+{\left({\vec{z}}^{i\to j}\right)}^{t}\\ & =\left\lfloor \frac{q}{2}\right\rfloor {\vec{\mu }}^{it}+{\vec{e}}^{it}\left(\vec{u}|-A^{j}-X^{i\to j}\right)+{\vec{\sigma }}^{t} \end{array}$$

where ${\vec{\sigma }}^{t}={\vec{y}}^{it}\left[\begin{array}{cc} 1 & 0_{1\times m}\\ 0 & R^{i\to j} \end{array}\right]+{\left({\vec{z}}^{i\to j}\right)}^{t}$ by (5), (6). Thus,

(7) $$\begin{array}{cc} ct^{j}\left(1;{\vec{s}}^{j}\right) & =\left(\left\lfloor \frac{q}{2}\right\rfloor {\vec{\mu }}^{it}+{\vec{e}}^{it}\left(\vec{u}|-A^{j}-X^{i\to j}\right)+{\vec{\sigma }}^{t}\right)\left(1;{\vec{s}}^{j}\right)\\ \; & =\left\lfloor \frac{q}{2}\right\rfloor \mu^{i}+{\vec{e}}^{it}\left(\vec{u}-A^{j}{\vec{s}}^{j}-X^{i\to j}{\vec{s}}^{j}\right)+{\vec{\sigma }}^{t}\left(1;{\vec{s}}^{j}\right)\\ \; & =\left\lfloor \frac{q}{2}\right\rfloor \mu^{i}+\underset{y}{\underset{︸}{{\vec{e}}^{it}\left(-X^{i\to j}{\vec{s}}^{j}\right)+{\vec{\sigma }}^{t}\left(1;{\vec{s}}^{j}\right)}} \end{array}$$

So we have the following Lemma 9.

**Lemma** **9.** *Let q,k,m,n be parameters for the above basic encryption scheme, χ be B-bounded. Set $\left({\vec{s}}^{j},A^{j}\right)\leftarrow HPRE.KeyGen\left(pp\right)$, $ct^{i}\leftarrow HPRE.Enc\left(pp,pk^{i},\mu^{i}\right)$, $ct^{j}\leftarrow HPRE.ReEnc\left(pp,rk^{i\to j},ct^{i}\right)$. Then*

$$ct^{j}\left(1;{\vec{s}}_{0}^{j}\right)=y+\left\lfloor \frac{q}{2}\right\rfloor \mu^{i},$$

*where $y=\left({\vec{y}}^{it}\left[\begin{array}{cc} 1 & 0_{1\times m}\\ 0 & R^{i\to j} \end{array}\right]+{\left({\vec{z}}^{i\to j}\right)}^{t}\right)\left(1;{\vec{s}}^{j}\right)+{\vec{e}}^{it}\left(-X^{i\to j}{\vec{s}}^{j}\right)$. Since $R^{i\to j}\leftarrow$SamplePre$\left(A^{i},T^{i},A^{j}+X^{i\to j}\right)$, we have $∥R^{i\to j}{∥}_{\infty }\leq B$ by Lemma 3. If $∥y∥\leq \left(m+1\right)\left(mB+1\right)B^{2}+nmB^{3}<\left\lfloor \frac{q}{2}\right\rfloor /2$, then μ←E.Dec(pp,sk,ct).*

Next, we consider the homomorphic operations of ciphertexts (including original ciphertexts and re-encryption ciphertexts). According to Lemma 9, the decryption of re-encryption ciphertext has the same form as the original ciphertext. Therefore, Lemma 8 shows that the homomorphism operation is feasible, including the homomorphic operation over the original ciphertexts, the homomorphic operation over the original ciphertexts and the re-encryption ciphertexts, and the homomorphic operation over the re-encryption ciphertexts. In addition, it is noted that the re-encryption ciphertexts has a larger decryption noise magnitude. Therefore, in order to prove that the HPRE scheme is L homomorphic, we only need to control the decryption noise magnitude of the homomorphic operations over the re-encryption ciphertexts. So similar to Theorem 1, we have Theorem 2.

**Theorem** **2.** *Let q,k,m,n,L be parameters for the above HPRE-SAC scheme, χ be B-bounded. If ${\left(O(mlogq)\right)}^{L+O(1)}<q/B^{3}$, then the HPRE-SAC scheme is L homomorphic.*

**Proof.**  Let $E_{i}$ be the bound of noise after evaluation on the i−th level of gates in ciphertext. By Lemma 9, we have $E_{0}\leq \left(m+1\right)\left(mB+1\right)B^{2}+nmB^{3}=O\left(m^{2}\right)B^{3}$. According to Lemma 8, when $mlog^{2}qB\leq E$ holds at a certain point, then $E_{i+1}=O\left(mlogq\right)\cdot E_{i}$ and $E_{L}={\left(O(mlogq\right)}^{L+O(1)}\cdot B^{3}$. Therefore, the decryption is correct if $E_{L}<\left\lfloor \frac{q}{2}\right\rfloor /2$, that is ${\left(O(mlogq)\right)}^{L+O(1)}<q/B^{3}$. □

Finally, we show that the HPRE-SAC scheme is multi-hop.

**Theorem** **3.** *Let q,k,m,n,L be parameters for the above HPRE-SAC scheme, χ be B-bounded, then the HPRE-SAC scheme is multi-hop.*

**Proof.**  Let the public key of user *i* be $pk^{i}=\left(\vec{u}|A^{i}\right)$, the re-encryption key from user *i* to user *j* be $rk^{i\to j}=R^{i\to j}$, i=1,2,⋯,l, the ciphertext of user 1 be $ct^{1}={\vec{e}}^{1t}\left(\vec{u}|-A^{1}\right)+{\vec{y}}^{1t}+\left\lfloor \frac{q}{2}\right\rfloor {\vec{\mu }}^{1t}$, where ${\vec{\mu }}^{1t}=\left(\mu^{1},0,\cdots ,0\right)$, ${\vec{e}}^{1}\leftarrow \chi^{1\times n}$, ${\vec{y}}^{1}\leftarrow \chi^{1\times m+1}$. If $ct^{i+1}\leftarrow HPRE.ReEnc\left(pp,rk^{i\to i+1},ct^{i}\right)$, then by (6), we have

$$ct^{2}=ct^{1}\left[\begin{array}{cc} 1 & 0_{1\times m}\\ 0 & R^{1\to 2} \end{array}\right]+{\left({\vec{z}}^{1\to 2}\right)}^{t},$$

$$\begin{array}{ccc} ct^{3} & =ct^{2}\left[\begin{array}{cc} 1 & 0_{1\times m}\\ 0 & R^{2\to 3} \end{array}\right]+{\left({\vec{z}}^{2\to 3}\right)}^{t}\\ & =ct^{1}\left[\begin{array}{cc} 1 & 0_{1\times m}\\ 0 & R^{1\to 2}R^{2\to 3} \end{array}\right]+{\left({\vec{z}}^{1\to 2}\right)}^{t}\left[\begin{array}{cc} 1 & 0_{1\times m}\\ 0 & R^{2\to 3} \end{array}\right]+ & {\left({\vec{z}}^{2\to 3}\right)}^{t} \end{array}$$

⋯

$$\begin{array}{cc} ct^{l} & =ct^{1}\left[\begin{array}{cc} 1 & 0_{1\times m}\\ 0 & \prod_{i=1}^{l-1}R^{i\to i+1} \end{array}\right]+\underset{{\vec{\rho }}_{1}^{t}}{\underset{︸}{\sum_{j=1}^{l-2}{\left({\vec{z}}^{j\to j+1}\right)}^{t}\left[\begin{array}{cc} 1 & 0_{1\times m}\\ 0 & \prod_{i=j+1}^{l-1}R^{i\to i+1} \end{array}\right]+{\left({\vec{z}}^{(l-1)\to l}\right)}^{t}}} \end{array}$$

By (5), we get

$$\begin{array}{cc} ct^{1}\left[\begin{array}{cc} 1 & 0_{1\times m}\\ 0 & \prod_{i=1}^{l-1}R^{i\to i+1} \end{array}\right] & =\left({\vec{e}}^{1t}\left(\vec{u}|-A^{1}\right)+{\vec{y}}^{1t}+\left\lfloor \frac{q}{2}\right\rfloor {\vec{\mu }}^{1t}\right)\left[\begin{array}{cc} 1 & 0_{1\times m}\\ 0 & \prod_{i=1}^{l-1}R^{i\to i+1} \end{array}\right]\\ & ={\vec{e}}^{1t}\left(\vec{u}|-A^{1}\prod_{i=1}^{l-1}R^{i\to i+1}\right)+\left\lfloor \frac{q}{2}\right\rfloor {\vec{\mu }}^{1t}+\underset{{\vec{\rho }}_{2}^{t}}{\underset{︸}{{\vec{y}}^{1t}\left[\begin{array}{cc} 1 & 0_{1\times m}\\ 0 & \prod_{i=1}^{l-1}R^{i\to i+1} \end{array}\right]}}\\ & ={\vec{e}}^{1t}\left(\vec{u}|-A^{l}-Y\right)+\left\lfloor \frac{q}{2}\right\rfloor {\vec{\mu }}^{1t}+{\vec{\rho }}_{2}^{t}, \end{array}$$

where $Y=X^{(l-1)\to l}+\sum_{j=1}^{l-1}X^{j\to (j+1)}\prod_{i=j+1}^{l-1}R^{i\to (i+1)}$. Therefore,

$$\begin{array}{cc} ct^{l}\left(1;{\vec{s}}^{l}\right) & =\left({\vec{e}}^{1t}\left(\vec{u}|-A^{l}-Y\right)+\left\lfloor \frac{q}{2}\right\rfloor {\vec{\mu }}^{1t}+{\left({\vec{\rho }}_{1}+{\vec{\rho }}_{2}\right)}^{t}\right)\left(1;{\vec{s}}^{l}\right)\\ & =\left\lfloor \frac{q}{2}\right\rfloor \mu^{1}\underset{\rho }{\underset{︸}{-{\vec{e}}^{1t}Y{\vec{s}}^{l}+{\left({\vec{\rho }}_{1}+{\vec{\rho }}_{2}\right)}^{t})\left(1;{\vec{s}}^{l}\right)}} \end{array}$$

If $∥\rho ∥=O\left(m^{l}\right)B^{l+1}<\left\lfloor \frac{q}{2}\right\rfloor /2$, the re-encryption ciphertext $ct^{l}$ can be correctly decrypted.

Similar to the proof of Lemma 9 and Theorem 2, we know that if ${\left(O(mlogq)\right)}^{l+L+O(1)}<q/B^{l+1}$, the HPRE-SAC scheme is multi-hop. □

### 5.3. Security Analysis

We show the security in this subsection.

**Theorem** **4.** *Let q,k,m,n,L be parameters for the above HPRE-SAC scheme, χ be B-bounded. If ${\left(O(mlogq)\right)}^{L+O(1)}<q/B^{3}$, then the HPRE-SAC scheme is IND-CPA secure based on LWE.*

**Proof.**  We consider the following games.

Game $G_{0}^{b}$: This game is the original game $Expt_{HPRE,A}^{CPA}\left(k\right)$ between challenger and adversary. Suppose that the index of target honest user is 0, the $pk^{0}=\left(\vec{u}|-A^{0}\right)$, $sk^{0}=\left({\vec{s}}_{L}^{0},T^{0}\right)$, $evk^{0}={\left\{P_{(l-1):l}^{0}\right\}}_{l=1,2,\cdots ,L}$, where $P_{(i-1):i}^{0}\leftarrow SwitchKeyGen\left({\vec{s}}_{l-1}^{0*},{\vec{s}}_{l}^{0}\right)$, ${\vec{s}}_{l}^{0*}=BD\left(1;{\vec{s}}_{l}^{0}\right)⊗BD\left(1;{\vec{s}}_{l}^{0}\right)$, ${\vec{s}}_{l}^{0}\leftarrow \chi^{m}$, ${\vec{s}}_{0}^{0}\leftarrow$SamplePre$\left(A,T,\vec{u}\right)$. The challenger computes the challenge ciphertext on query μ as follows:

- If b=0, it returns $ct={\vec{e}}^{0t}\left(\vec{u}|-A^{0}\right)+{\vec{y}}^{0t}+\left\lfloor \frac{q}{2}\right\rfloor {\vec{\mu }}^{t}$, where ${\vec{\mu }}^{t}=\left(\mu ,0,\cdots ,0\right)$, ${\vec{e}}^{0}\leftarrow \chi^{1\times n}$, ${\vec{y}}^{0}\leftarrow \chi^{1\times m+1}$.
- If b=1, it returns a random ciphertext $ct\leftarrow Z_{q}^{1\times m+1}$

Game $G_{1}^{b}$: We modify the encryption key generation oracle $O_{pk}$. This game is identical to game $G_{0}$, except that the challenger replaces $A^{i}$ of user *i* with $A_{+}^{i}$, where $(A_{+}^{i},T_{+}^{i})\leftarrow$ TrapGen(q,n,m).

Because of $(A_{+}^{i},T_{+}^{i})\leftarrow$TrapGen(q,n,m), $(A^{i},T^{i})\leftarrow$ TrapGen(q,n,m), we have $A_{+}^{i},A^{i}$ are statistically close to uniform by Lemma 1. Therefore, $A_{i}\approx_{s}A_{i}^{+}$. So $G_{0}^{b}\approx_{s}G_{1}^{b}$

Game $G_{2}^{b}$: We modify the evaluation key generation oracle $O_{evk}$. The challenger computes $P_{(l-1):l,+}^{i}\leftarrow SwitchKeyGen\left({\vec{s}}_{l-1+}^{i*},{\vec{s}}_{l+}^{i}\right)$, where ${\vec{s}}_{l+}^{i*}=BD\left(1;{\vec{s}}_{l+}^{i}\right)⊗BD\left(1;{\vec{s}}_{l+}^{i}\right)$, ${\vec{s}}_{0+}^{i}\leftarrow$SamplePre$\left(A_{+}^{i},T_{+}^{i},\vec{u}\right)$, ${\vec{s}}_{l+}^{i}\leftarrow \chi^{m}$, and replaces $P_{(l-1):l}^{i}$ of user *i* with $P_{(l-1):l,+}^{i}$, l=1,2,⋯,L. The rest are the same as $G_{1}^{b}$.

Since ${\vec{s}}_{L}^{i}\leftarrow \chi^{m}$$\left({\vec{s}}_{L+}^{i}\leftarrow \chi^{m}\right)$ is only used to generate $P_{(L-1):L}^{i}$$\left(P_{(L-1):L,+}^{i}\right)$, we can get $P_{(L-1):L}^{i}$$\left(P_{(L-1):L,+}^{i}\right)$ indistinguishable from uniform by Lemma 7. Therefore, $P_{(L-1):L}^{i}\approx_{s}P_{(L-1):L,+}^{i}$.Then we can get $P_{(l-1):l}^{i}\approx_{s}P_{(l-1):l,+}^{i}$ in descending order, l=1,2,⋯,L. So $G_{1}^{b}\approx_{s}G_{2}^{b}$.

Game $G_{3}^{b}$: We modify the re-encryption key generation oracle $O_{rk}$. the challenger samples $R_{+}^{i\to j}\leftarrow \chi^{m\times m}$ and replaces $R_{i\to j}$ with $R_{+}^{i\to j}$. The rest are the same as $G_{2}^{b}$.

Because of $A^{i}R^{i\to j}=A^{j}+X^{i\to j}$, we have $A^{1}R^{1\to 2}R^{2\to 3}=\left(A^{2}+X^{1\to 2}\right)R^{2\to 3}=A^{3}+X^{2\to 3}+X^{1\to 2}R^{2\to 3}$. Therefore, the adversary cannot use $R^{1\to 2},R^{2\to 3}$ to verify the relationship between $A^{1}$$A^{2}$ and $A^{3}$. So $R^{i\to j}$ is independent of each other. Since $R^{i\to j}\leftarrow$SamplePre($A^{i},T^{i},A^{j}+X^{i\to j}$), we know $R^{i\to j}$ statistically close to $\chi^{m\times m}$ by Lemma 3.That is $R_{i\to j}\approx_{s}R_{+}^{i\to j}$. So $G_{2}^{b}\approx_{s}G_{3}^{b}$.

Game $G_{4}^{b}$: We modify re-encryption ciphertext generation oracle $O_{re}$. The challenger replaces the re-encrypted ciphertext $ct^{j}$ with $ct_{+}^{j}\leftarrow$ HPRE.ReEnc $\left(pp,r^{i\to j},ct^{i}\right)$. The rest are the same as $G_{3}^{b}$.

According to Lemma 3, we have the $R_{i\to j}\approx_{s}R_{i\to j}^{+}$. It follows that $G_{3}^{b}\approx_{s}G_{4}^{b}$, for efficient adversary.

Finally, we have that $G_{4}^{1}\approx_{c}G_{4}^{0}$ from LWE. Combining the above indistinguishability, we have shown that $G_{0}^{1}\approx_{c}G_{0}^{0}$. This completes the proof. □

It should be noted that our HPRE-SAC scheme uses trapdoor to generate re-encryption key and decryption key respectively, which not only ensures the homomorphism, but also ensures the resistance to strong collusion attack. By Lemma 4, we know that the trapdoor sampling algorithm is one-way and collision-resistant, so the delegatee and the proxy can not attack the decryption key of the delegator. In addition, the decryption key does not participate in the re-encryption key generation, and is only used for ciphertext decryption. Therefore, the adversary can not get any information of the decryption key, so the approximate value of the decryption key can not be obtained.

If the adversary obtains the approximate value $P2\left(\vec{s}\right)+\vec{x}$ of the decryption key $\vec{s}$, where $\vec{x}$ is an error distribution, then the adversary can decrypt the delegator’s ciphertext. Let $ct=\left(ct_{1},ct_{2}\right)=\left({\vec{e}}^{t}\vec{u}+y+\left\lfloor \frac{q}{2}\right\rfloor \mu ,{\vec{e}}^{t}\left(-A\right)+{\vec{y}}^{t}\right)$, then we have

$$\begin{array}{cc} \left(ct_{1},BD\left(ct_{2}\right)\right)\left(1;P2\left(\vec{s}\right)+\vec{x}\right) & =ct_{1}+ct_{2}\vec{s}+BD\left(ct_{2}\right)\vec{x}\\ & =\left(ct_{1},ct_{2}\right)\left(1;\vec{s}\right)+BD\left(ct_{2}\right)\vec{x}\\ & =\left({\vec{e}}^{t}\left(\vec{u}|-A\right)+{\vec{y}}^{t}+\left\lfloor \frac{q}{2}\right\rfloor {\vec{\mu }}^{t}\right)\left(1;\vec{s}\right)+BD\left(ct_{2}\right)\vec{x}\\ & ={\vec{e}}^{t}\left(\vec{u}|-A\right)\left(1;\vec{s}\right)+{\vec{y}}^{t}\left(1;\vec{s}\right)+\left\lfloor \frac{q}{2}\right\rfloor {\vec{\mu }}^{t}\left(1;\vec{s}\right)+BD\left(ct_{2}\right)\vec{x}\\ & =\underset{x}{\underset{︸}{{\vec{y}}^{t}\left(1;\vec{s}\right)+BD\left(ct_{2}\right)\vec{x}}}+\left\lfloor \frac{q}{2}\right\rfloor \mu . \end{array}$$

If ${∥x∥}_{\infty }<\left\lfloor \frac{q}{2}\right\rfloor /2$, then the decryption is correct. Thus, the IND-CPA security of the HPRE-SAC scheme does not hold, which is in contradiction with Theorem 4. Therefore, the adversary can not obtain the approximate value $P2\left(\vec{s}\right)+\vec{x}$ of the decryption key $\vec{s}$.

In addition, although our HPRE-SAC scheme is single bit encryption, we can use homomorphic ciphertext packing technology [51] and trapdoor based multi bit proxy re-encryption scheme [27] to construct a multi bit homomorphic proxy re-encryption scheme against strong collusion attack.

### 5.4. Comparisons

We compare the related works in this subsection.

At present, there are many PRE schemes. We only select some related works from the lattice based PRE and compare them with our schemes. It can be seen from Table 1 that Ma et al. [28], Li et al. [44,45], Li et al. [46] and our scheme are homomorphic proxy re-encryption schemes. The following comparison is made from the length of the encryption key, decryption key, re-encryption key and ciphertext (including original ciphertexts and re-encryption ciphertexts). The comparison results are shown in Table 3.

It can be seen from Table 3 that the public key length of Ma et al. [28] is nlogq, that of Li et al. [44] is m(n+1)logq, that of Li et al. [45] is the same as that of Li et al. [44], and that of Li et al. [46] is the longest, which is (nlogn+2)logq. The length of the public key of our HPRE-SAC scheme is nm, which is smaller than that of Li et al. [44] and only one constant times different from that of Ma et al. [28]. From the length of re-encryption key, we can find that the complexity of Ma et al. [28] is $O(n^{3}logq)$, that of Li et al. [46] is only O(nlogq), and the rest is $O(n^{2}logq)$. However, by observing the length of the ciphertext (including original ciphertexts and re-encryption ciphertexts), we can find that the length of the ciphertext of Li et al. [46] is the largest, that is $O({\left(nlogq\right)}^{2}logq)$, while that of our scheme HPRE-SAC and [44,45] are the smallest, the complexity is only O(nlogq). In conclusion, the comparison shows that our scheme HPRE-SAC has better parameters. In addition, it should be noted from Table 1 that only our HPRE-SAC scheme can resist strong collusion attack.

### 5.5. An Application

In this section, we present an application of our scheme HPRE-SAC: Secure computing of personal healthcare records (PHRs) in the cloud.

**Figure 3:**
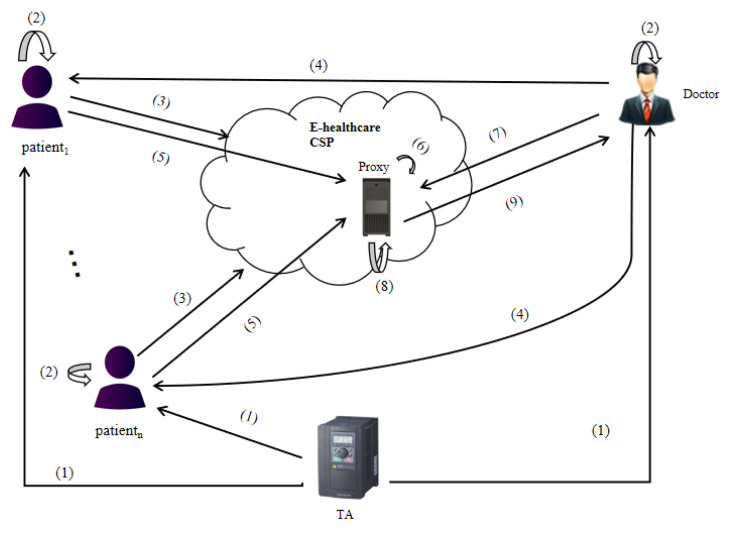
Secure computing of personal healthcare records (PHRs) using HPRE with strong anti-collusion (SAC) in the cloud.

At present, there are many applications of PRE in the cloud [52,53,54,55,56], especially in cloud based PHRs [57,58]. The overall system architecture of cloud based PHRs computing using the proposed HPRE-SAC scheme is shown in Figure 3. It includes four entities: patient (data owner), E-Healthcare cloud service provider (CSP), trusted authority (TA) and doctor (data receiver). The following steps are required.

(1) Patients and the doctor use the algorithm HPRE.Setup to register in TA to obtain the public parameters of the system.
(2) Patients and the doctor use the algorithm HPRE.KeyGen to generate their own encryption key, public evaluation key and decryption key.
(3) Patients use the algorithm HPRE.Enc to encrypt their PHRs and upload them to the E-healthcare cloud service provider for storage. The PHRs here includes not only diagnostic information from doctors, but also personal health information collected by smart wearable devices. We assume that the E-healthcare cloud service provider is not trusted, so the patients need to encrypt the data.
(4) For a certain purpose (in addition to clinical purposes, it can also be for research purposes), the doctor asks patients for the right to decrypt their encrypted data.
(5) After the patient agrees with the doctor’s request, the algorithm HPRE.ReKey is used to generate the re-encryption key and send it to the proxy.
(6) Suppose that the proxy residing in the cloud is semi-trusted, that is to say, it follows the protocol, but can collect information to infer private information, or collude with the data user to attack the data owner. The proxy re-encrypts the patient’s ciphertext to generate the doctor’s ciphertext by using the algorithm HPRE.ReEnc.
(7) The doctor needs to analyze and calculate the PHRs of multiple patients for a certain purpose (in addition to clinical purpose, it can also be for research). In order to reduce the burden of local computation and communication, the doctor sends the function to the proxy.
(8) The proxy uses the algorithm HPRE.Eval to perform homomorphic function operation on the re-encryption ciphertext belonging to the doctor.
(9) The doctor downloads the results of homomorphic operation and decrypts them locally by using the algorithm HPRE.Dec to obtain the required data.

In this system architecture, it not only ensures the safety of the patient’s data, but also meets the efficient needs of doctors for the statistical analysis of PHRs of multiple patients.

## 6. Conclusions

In order to adapt to efficient and secure cloud computing, this paper proposes a lattice based homomorphic proxy re-encryption scheme, namely HPRE-SAC, which can resist strong collusion attack. In particular, the HPRE-SAC scheme is unidirectional, multi-hop, and CPA secure under LWE. Compared with the existing HPRE scheme, the HPRE-SAC scheme has better parameters. However, the efficiency of the HPRE-SAC scheme is still low. The future work will be to construct a more efficient HPRE scheme based on the existing scheme, such as constructing an HPRE scheme on the ring LWE to meet the more comprehensive application requirements.

## Figures and Tables

**Table 1:** Comparison of lattice-based proxy re-encryption (PRE) schemes.

Scheme	LWE	Security	Hop	Direction	HE	Anti-Collusion
Li et al. [20]	Y	CPA	Single	Uni-	N	weak
Singh et al. [25]	Y	CPA	Single	Uni-	N	weak
Xagawa [29]	Y	N	Single	Uni-	N	weak
Kirshanova [33]	Y	CCA	Single	Uni-	N	strong
Nishimaki et al. [34]	Y	CPA	Single	Uni-	N	weak
Hou et al. [35]	Y	CPA	Multi	Bi-	N	weak
Yin et al. [36]	Y	CPA	Single	Uni-	N	strong
Yin et al. [37]	Y	CPA	Single	Uni-	N	strong
Jiang et al. [26]	Y	CPA	Multi	Uni-	N	strong
Ma et al. [28]	Y	CPA	Single	Uni-	Y	weak
Li et al. [44]	Y	CPA	Multi	Uni-	Y	weak
Li et al. [45]	Y	CPA	Multi	Uni-	Y	weak
Li et al. [46]	Y	CPA	Single	Uni-	Y	weak
Our Scheme FHPR-SAC	Y	CPA	Multi	Uni-	Y	strong

Y indicates that the scheme has been achieved and N indicates that the scheme has not been achieved.

**Table 2:** Notation.

*x*	scalar
⌊x⌉	rounding *x* to the nearest integer
$\left\lfloor x\right\rfloor (\left\lceil x\right\rceil$)	rounding down (up)
$\vec{x}$	vector
*A*	matrix or set
$\left|\right|\vec{x}{\left|\right|}_{p}$	$l_{p}$ norm of $\vec{x}$
$P2\left(\vec{x}\right)$	$\left(1\vec{x};2\vec{x};\cdots ;2^{\lceil logq\rceil -1}\vec{x}\right)\in Z_{q}^{n\lceil logq\rceil }$, where $\vec{x}\in Z_{q}^{n}$
$BD\left(\vec{x}\right)$	$\left({\vec{u}}_{1},\cdots ,{\vec{u}}_{\left\lceil logq\right\rceil }\right)\in {\left\{0,1\right\}}^{n\lceil logq\rceil }$, where $\vec{x}=\sum_{k=1}^{\left\lceil logq\right\rceil }2^{k-1}{\vec{u}}_{k}$
(X|Y)	the concatenation of the columns of X,Y
(X;Y)	the concatenation of the rows of X,Y
x←χ	*x* is sampled according to a probability distribution χ
x←S	*x* is sampled uniformly from a set S
$X\approx_{c}Y$	*X* and *Y* are computationally indistinguishable
$X\approx_{s}Y$	*X* and *Y* are statistically indistinguishable

**Table 3:** The parameters comparison of HPRE schemes.

Scheme	∥pk∥	∥sk∥	∥rk∥	∥ct∥
Ma et al. [28]	nlogq	*n*	$n^{3}logq+n^{2}logq$	$n^{2}logq$
Li et al. [44]	m(n+1)logq	(2m+1)logq	(2n+1)((2n+1)logq+m)	(2n+1)logq
Li et al. [45]	m(n+1)logq	(n+1)logq	(n+1)((n+1)logq+m)logq	(n+1)logq
Li et al. [46]	(nlogn+2)logq	nlogn	nlogq+2	${\left(nlogq+2\right)}^{2}log+nlog\left(n+2\right)$
HPRE-SAC	nm	*m*	${\left(m+1\right)}^{2}$	m+1

∥pk∥, ∥sk∥, ∥rk∥, ∥ct∥ represent the length of encryption key, decryption key re-encryption key and ciphertext (including original ciphertexts and re-encryption ciphertexts) respectively, m=6n⌈logq⌉.
